# What is the role of randomised trials in implementation science?

**DOI:** 10.1186/s13063-023-07578-5

**Published:** 2023-08-16

**Authors:** Robbie Foy, Noah M. Ivers, Jeremy M. Grimshaw, Paul M. Wilson

**Affiliations:** 1 Leeds Institute of Health Sciences, University of Leeds, Leeds, UK; 2 Women’s College Hospital, Department of Family and Community Medicine, University of Toronto, Toronto, Canada; 3 Ottawa Hospital Research Institute, Ottawa, Canada; 4 Centre for Primary Care and Health Services Research, University of Manchester, Manchester, UK

**Keywords:** Cluster randomised controlled trials, Implementation science, Quality improvement

## Abstract

**Background:**

There is a consistent demand for implementation science to inform global efforts to close the gap between evidence and practice. Key evaluation questions for any given implementation strategy concern the assessment and understanding of effects. Randomised trials are generally accepted as offering the most trustworthy design for establishing effectiveness but may be underused in implementation science.

**Main body:**

There is a continuing debate about the primacy of the place of randomised trials in evaluating implementation strategies, especially given the evolution of more rigorous quasi-experimental designs. Further critiques of trials for implementation science highlight that they cannot provide ‘real world’ evidence, address urgent and important questions, explain complex interventions nor understand contextual influences. We respond to these critiques of trials and highlight opportunities to enhance their timeliness and relevance through innovative designs, embedding within large-scale improvement programmes and harnessing routine data.

Our suggestions for optimising the conditions for randomised trials of implementation strategies include strengthening partnerships with policy-makers and clinical leaders to realise the long-term value of rigorous evaluation and accelerating ethical approvals and decluttering governance procedures for lower risk studies.

**Conclusion:**

Policy-makers and researchers should avoid prematurely discarding trial designs when evaluating implementation strategies and work to enhance the conditions for their conduct.

## Background

Gaps between evidence and practice pervade different healthcare systems and specialties [1–7]. They are associated with poorer patient outcomes [3–5, 8] and can be resistant to change [6, 9, 10]. There is over-treatment as well as under-treatment [11, 12]. During the COVID-19 pandemic, access to medical interventions such as vaccines was inconsistently matched to population need [13].

These research-to-practice gaps represent a strategically important problem for policy-makers, healthcare systems and research funders because they limit the health, social and economic impacts of research [14]. Regular processions of policy reforms aim to tackle inappropriate variations to ensure the quality and safety of healthcare. Their effects, efficiency and reach frequently fall short of initial hopes and expectations [9, 15, 16]. Indeed, there are serious risks of wasted effort if urgent drives to improve care are insufficiently underpinned by scientific methods [17]. There is therefore a predictable and sustained need for implementation science, which aims to inform policy decisions about how best to use resources to improve the uptake of research findings by evaluating approaches to change clinical and organisational behaviour [18]. Such approaches may include interventions such as audit and feedback (providing a summary of clinical performance over a specified period of time), computerised decision support systems and financial incentives [19]. Implementation interventions typically aim to increase uptake of clinical interventions of established effectiveness (e.g. intensification of treatment for type 2 diabetes [20]) or reduce use of low value or harmful clinical interventions (e.g. x-rays for non-specific low back pain [21]).

There are key evaluation questions of interest to patients, professionals and policy-makers for any given implementation strategy. Does it work? For which contextual features and targeted behaviours is it likely to work? Are the costs worth the benefits?

Well-conducted randomised controlled trials offer a ‘fair test’ of effectiveness by balancing known and unknown confounders so that differences in outcomes between comparison groups can be confidently attributed to intervention effects. Given that implementation strategies usually target organisations, cluster randomisation (e.g. of general practices or hospitals) is usually more appropriate than individual patient randomisation [22, 23]. Across health systems, there is a growing impetus to innovate, experiment and rapidly implement new solutions for health care problems—this was especially true during the COVID-19 pandemic. However, there is a continuing debate about the place of trials in the field of implementation science [24–28], especially given that many strategies to change professional and organisational behaviours can be conceptualised as complex interventions [29].

We respond to critiques of trials for implementation science and highlight opportunities to enhance their design, delivery and efficiency. We suggest that policy-making and research communities work to optimise the conditions for conducting trials.

## Main text

### Critiques of trials

#### Alternative evaluation designs offer similar protections against bias

There are natural, human tendencies to look for expected or even hoped for intervention effects, and hence reach erroneous conclusions from an evaluation. Comparisons of more and less rigorous evaluation methods suggest that the best way to show that an intervention ‘works’ is to use a weak non-randomised (quasi-experimental) study design without a concurrent control group [30, 31]. However, more rigorous quasi-experimental evaluation designs offer a viable alternative to trials for evaluating implementation strategies [32–35]. For example, a reanalysis of cluster randomised trials as interrupted time series found that effect estimates were largely concordant [36]. Rigorous quasi-experimental studies can make important policy contributions where randomisation was not acceptable or feasible, for example, in examining the effects of withdrawing financial incentives on adherence to primary care indicators [37].

Whilst relatively sophisticated quasi-experimental designs hold considerable promise, further experience is needed to understand their properties, strengths and limitations in much the same way that it has taken decades to develop evidence-informed criteria to judge the validity and generalisability of trials [38]. Such criteria also guide researchers to anticipate and implement established measures to reduce observational and performance biases.

The understanding of known and unknown confounders within the context of healthcare systems is relatively poor compared to, say, biological systems. Multiple contextual influences on the outcomes of implementation strategies [39] make attribution of intervention effects challenging, if not heroic, in the absence of rigorously controlled evaluations. Non-randomised designs may not be able to frame or rule out plausible rival hypotheses to any apparent intervention effect, particularly within the complex and evolving contexts of healthcare systems. The interpretation of changes over time is particularly vulnerable to system-level disruptions, the COVID-19 pandemic being an extreme case.

Confident attribution is important given that the observed effect sizes of implementation interventions can be small, even if worthwhile from population perspectives. For example, a trial of a multifaceted implementation strategy found just over a 1% absolute difference in high-risk prescribing between intervention and control general practices, which translated into a cost-effective population benefit from reduced patient harm [40].

#### Trials cannot provide ‘real world’ evidence

Clinical trials are dogged by the criticism that they recruit highly selected participants atypical of wider patient populations who may also receive above average attention, thereby limiting generalisability [41]. Such a criticism could justly be levelled at an implementation trial that recruits selected sites and delivers interventions requiring resources and skillsets not typically available to healthcare systems. Yet, most implementation trials tend to be pragmatic in several ways [42], by recruiting unselected sites, adapting available interventions, allowing flexibility of delivery and using non-intrusive data collection to assess outcomes [40]. For example, Elouafkaoui et al. randomly allocated all 795 National Health Service general dental practices in Scotland to receive or not to receive individualised feedback, derived from routinely collected data, and found that feedback reduced the antibiotic prescribing rate by 5.7% [43]. In contrast, less rigorous effectiveness evaluations may include relatively small numbers of volunteer sites with limited generalisability [22, 44].

#### Trials cannot address urgent and important questions

Almost every healthcare problem, such as avoidable cancer deaths and strokes or maximising COVID vaccination coverage, demands urgent solutions. The history of medicine is littered with obsolete clinical recommendations based on partial evidence and assumptions, subsequently overturned by rigorous studies [45–49]. Similarly, major and costly initiatives have ended or been threatened because of continuing uncertainty over their benefits because trials were considered unacceptable and unfeasible [50–52]; given the cyclical nature of policy reforms [53], it is likely that similar initiatives will emerge again and be under-evaluated again.

Alternative evaluation designs offer the attraction of faster turnaround times than trials, with shorter planning and follow-up periods. However, time series designs depend upon stable data being available over lengthy periods. Some of the long timelines associated with implementation trials are related to burdensome research regulation and management [54], a limitation of the wider system rather than trials per se.

Well-conducted trials can overturn conventional wisdom whilst positive quasi-experimental studies may fail to convince. A trial of a multidisciplinary intervention targeting evidence-based management of fever, hyperglycaemia and swallowing dysfunction in acute stroke units found significant reductions in patient deaths or dependency at 90 days [55] and in deaths after 4 years [56]. The clinical benefits were markedly greater than those observed for other interventions, such as stroke units or thrombolysis. It might otherwise have been more difficult to convince sceptics of the value of the multidisciplinary intervention.

#### Trials shed little light on complex interventions

There are challenges in evaluating complex interventions, which contain several interacting components and target one or more behaviours, target more than one group or organisational level, result in different numbers and types of outcome and permit degrees of tailoring or flexibility [57]. Some of these interventions, such as digital health records and service configurations, may evolve over time and become outmoded before any evaluation is completed.

It has been suggested that whilst randomised trials may be appropriate for clinical interventions, ‘service innovations are even more complex, and this complexity needs to be embraced, not eliminated’ [27]. Hence, mixed method evaluations incorporating quasi-experimental designs are better suited to evaluating large-scale service reconfigurations, such as hyperacute stroke care [58]. However, alternative methods of evaluating complex interventions may have their own pitfalls. For example, multiple case studies are unlikely to include a robust assessment of whether any intervention works and unlikely to have a large and representative enough sample to allow generalizable conclusions.

Some complex interventions are not ready for trial evaluation. The UK Medical Research Council framework for the development and evaluation of complex interventions recommends the use of multiple research methods, with scope for embedding trials within a broader programme of studies which can also contribute to understanding mechanisms of change [29]. The framework further recognises the need for a sound theoretical understanding of causality (e.g. within a logic model) and hence the definition of prototypical elements followed by feasibility testing in context to help decide when an evolving intervention is stable enough for trial evaluation [59].

#### Trials shed little light on contextual influences

Contextual factors can have major influences on intervention effects. A criticism of trials is that by controlling for and ‘eliminating’ the influences of contextual factors, trials cannot provide information about their impacts on change [60]. This criticism may apply to any effectiveness evaluation. Trials can help understand contextual influences in three ways. First, they provide an opportunity to not only look at the mean effect but to explore whether contextual variations matter, with less concern about unknown confounders. For example, a trial in general practice demonstrated that antimicrobial stewardship, comprising a webinar, monthly feedback reports and electronic decision support, was effective for adults but not children, suggesting the need for an alternative approach for a different patient population [61]. Second, qualitative and quantitative process evaluations, ideally conducted in parallel to trials, also generate insights into contextual influences [62]. A process evaluation indicated that a multifaceted strategy to improve induced abortion care was ineffective because gynaecology teams were already highly motivated to follow best practice guidance but hindered by organisational constraints [63]. Third, comparing findings of similar interventions for different targeted behaviours or across different settings allows indirect comparisons of contextual modifiers, especially via systematic reviews. A meta-analysis of 122 trials of computerised clinical decision support systems found that low baseline adherence and paediatric settings were associated with significantly larger absolute improvements in care [64]. Thus, pursuing a rigorous answer to the question of ‘whether’ an implementation strategy worked is not mutually exclusive to—and may in fact facilitate—elaborations of theory regarding ‘where’, ‘how’, and ‘why’.

### Innovations and opportunities

#### Identifying and prioritising ‘best bet’ interventions

Implementation interventions typically have several components but conducting multiple trials of every permutation can be wasteful. For example, varying only five elements of audit and feedback (e.g. differing frequencies of feedback) produces 288 combinations—not allowing for replication of studies or the addition of other interventions, such as educational meetings or outreach visits [65]. Some trial designs allow for adaptation of intervention components or assignment to interventions as evaluations proceed.

The Multiphase Optimization Strategy (MOST) offers a methodological approach for building, optimising and evaluating multicomponent interventions. MOST comprises three steps: preparation, laying the groundwork for optimisation by conceptualising and piloting components; optimisation, conducting trials to identify the most promising single or combined intervention components; and evaluation, a definitive randomised trial to assess intervention effectiveness [66]. Modelling experiments can identify and prioritise the most promising ‘active ingredients’ for further study [67]. These experiments can be conducted virtually (e.g. online) with targeted participants using proxy outcomes (e.g. behavioural intentions) [68].

The Sequential Multiple Assignment Randomized Trial (SMART) allows identification of the best tailoring variables and uses decision rules for adaptive interventions based upon early findings. It is especially suited for building time-varying adaptive interventions. It has been used to tailor the intensity of an intervention to improve uptake of a re-engagement programme for patients with serious mental illness according to site characteristics and initial responses to interventions [69].

The stepped wedge design offers a solution where there is uncertainty, but randomisation to a non-intervention control is unacceptable. It entails introducing an intervention to groups of clusters in a random order. There are no ‘losers’ because all sites eventually receive the intervention. A stepped wedge trial demonstrated that an intervention comprising professional education, informatics to facilitate review and financial incentives reduced high-risk prescribing in general practices [70]. Stepped wedge trials can be complex to conduct [71] and their analysis fraught with pitfalls [72, 73]. One assumption, that the intervention does no harm, may not hold; a stepped wedge trial of predictive risk stratification to identify and manage patients at higher risk of emergency hospital admissions found that it increased emergency attendances, hospitalisation and costs without benefiting patients [74].

#### Implementation laboratories

Trials offer opportunities to optimise the effectiveness of existing implementation interventions, in much the same way that clinical research has continually pushed marginal gains in the effective management of conditions such as cancer or stroke. Yet, establishing the infrastructure for each new trial can be costly and time-consuming. There are opportunities for implementation researchers to learn from and adapt methodologies from clinical fields, such as oncology; innovations such as ‘master protocols’ are based upon a single overarching design to evaluate multiple hypotheses with the goal of improving efficiency and standardising the development and evaluation of different interventions [75].

Large-scale programmes offer opportunities for embedded trials. The PRevention of Cerebral Palsy in Pre-Term labour (PReCePT) programme aimed to reduce cerebral palsy by promoting the use of magnesium sulphate in pregnant women at risk of premature delivery in England. The programme included a nested trial comparing two approaches to quality improvement [76].

The next evolutionary step is to create a learning health system which makes small, incremental changes supported by tightly focused evaluations, and thereby cumulatively improves patient care whilst developing the underpinning evidence base. Such ‘radical incrementalism’ offers a potentially cost-effective if under-utilised approach to embedding learning within large scale improvement programmes [77]. It has already been used in public policy and in business [78]; *Amazon* and *eBay* randomise potential customers to different presentations of their products online to understand what drives purchases. It is also applicable to healthcare. For example, within a national clinical audit programme, is feeding back data on performance indicating an organisation’s position against the top 10% more likely to stimulate improvement than showing its position against median performance? Does adding quality improvement facilitation to standard feedback have effects over standard feedback alone? Implementation laboratories entail embedding a sequential programme of head-to-head trials testing different versions of interventions within an established improvement initiative [79, 80]. Those versions identified as more effective than the current standard become the new standard whilst those which are not more effective are discarded. The UK National Clinical Audit of Blood Transfusions collaborated in trials comparing different ways of presenting content and supporting delivery of feedback reports to hospitals [81]. Similar opportunities apply to other frequently-used implementation strategies, such as clinical decision support systems or educational programmes.

Implementation laboratories several further advantages. First, they can reduce research waste [82], such as the failure to build upon empirical findings in developing and evaluating feedback interventions [83]. Second, cluster randomised trials typically require larger numbers of patients than individually randomised trials to account for lack of independence at the cluster level. Increasing the number of sites generally buys greater statistical efficiency than increasing the number of patients. Embedding trials within an existing network or major improvement initiative facilitates recruitment, data collection and helps ensure ‘real world’ generalisability, building on the advantages of registry-based trials [84]. Third, comparing and integrating findings from different implementation laboratories through a ‘meta-laboratory’ allows learning about important contextual effect modifiers and mediators.

#### Harnessing routinely collected data

The collection and analysis of project–specific data is expensive and limits the volume and duration of data collection. Routinely collected data can be applied to develop quality indicators [85], analyse variations in care [12] and assess outcomes in trials of implementation strategies [86, 87]. Routine ‘big’ datasets offer opportunities to improve research efficiency, improve internal validity via non-intrusive data collection (and reduce risk of Hawthorne effects) and enhance generalisability and reach through participation of unselected healthcare provider and patient populations. For example, a trial of practice facilitation to support family physicians to engage with their patients around COVID-19 vaccinations used existing administrative databases to identify the practices with greatest need and allocated them to the offer of support or usual care [88].

Trials using routinely collected data may also be able to achieve relatively large sample sizes, hence bolstering statistical power to detect modest effect sizes and explore effect modifiers. However, larger samples may be needed to compensate for additional ‘noise’ from using data not originally intended for research. It is important to ensure that any routinely available data are a good fit for the outcomes of interest or based upon reasonable assumptions about relevance. Using unplanned hospital readmission rates in evaluating interventions to improve the process of hospital discharge to patient homes assumes that most such readmissions are driven by adverse events and would rather be avoided by patients and healthcare systems. Just as innovative ‘platform trials’ have been crucial to guide the clinical treatment of COVID-19 [89], custom-built registries used by national clinical audit programmes offer platforms for implementation trials [90]. They also provide a means for monitoring subsequent uptake and population impact of implementation strategies beyond trial lifetimes [91].

#### Optimising conditions for trials

Increasing burdens of research regulation and management may have unwittingly conspired to undermine the feasibility and timeliness of trials [54]. Experience of the COVID-19 pandemic has demonstrated that approvals and governance procedures can be streamlined with sufficient will [92, 93]. There are calls to make the conduct of trials for drug development easier, faster and cheaper [94]. There is an equally strong case for similar actions around lower-risk research which aims to accelerate the uptake of evidence-based practice. Table 1 suggests some avenues to explore in optimising the conditions for the conduct of implementation trials.

**Table 1:** Suggestions for optimising the conditions for randomised trials of implementation strategies

Anticipating the need to design and conduct trials should quasi-experimental evaluations show promising results insufficient to shift equipoise
Building partnerships with policy-makers and clinical leaders and promoting the long-term value of rigorous evaluation
Funding programmes of trials to evaluate progressive incremental changes to implementation strategies
Embedding the design and delivery of trials within large scale improvement programmes
Accelerating ethical approvals and decluttering governance procedures for lower risk studies
Reducing the burden and intrusiveness of outcome assessment by using routinely collected data

## Conclusion

Trials generally offer known protection against threats to internal validity, chiefly selection bias, in the evaluation of implementation strategies. Their findings are less dependent on skilled and nuanced interpretation compared to other study designs. Pragmatic trials can provide real world evidence in addressing important implementation problems and improve understanding of both complex interventions and contextual influences. There are opportunities to advance implementation science and its impact through innovative trial designs, implementation laboratories and the use of routine data. We encourage researchers, funders and policy-makers to consider when randomised evaluations would be feasible and preferable and work to optimise conditions for their conduct.

## Data Availability

Not applicable.
